# Hepatotoxicity Evaluation of Aqueous Extract from *Scutia buxifolia*

**DOI:** 10.3390/molecules18077570

**Published:** 2013-06-28

**Authors:** Robson Borba de Freitas, Bruno Tomazele Rovani, Aline Augusti Boligon, Thiele Faccim de Brum, Mariana Piana, Roberta da Silva Jesus, Carolina Fantinel Veloso, Helena Kober, Rafael Noal Moresco, Isabel Cristina da Costa Araldi, Liliane de Freitas Bauermann, Margareth Linde Athayde

**Affiliations:** 1 Graduate Program in Pharmaceutical Sciences, Center of Health Sciences, Federal University of Santa Maria, Santa Maria, RS, 97105-900, Brazil; 2 Graduate Program in Human Communication Disorders, Center of Health Sciences, Federal University of Santa Maria, Santa Maria, RS, 97105-900, Brazil; 3 Graduate Program in Pharmacology, Center of Health Sciences, Federal University of Santa Maria, Santa Maria, RS, 97105-900, Brazil; 4 Department of Clinical and Toxicological Analysis, Center of Health Sciences, Federal University of Santa Maria, Santa Maria, RS, 97105-900, Brazil; 5 Department of Physiology and Farmacology, Center of Health Sciences, Federal University of Santa Maria, Santa Maria, RS, 97105-900, Brazil; 6 Department of Industrial Pharmacy, Center of Health Sciences, Federal University of Santa Maria, Santa Maria, RS, 97105-900, Brazil

**Keywords:** *Scutia buxifolia*, hepatotoxicity, transaminases, redox status, HPLC/DAD

## Abstract

Nowadays there is an increase in the number of people taking herbals worldwide. *Scutia buxifolia* is used for the treatment of hypertension, but little is known about its action on liver. Thirty-two Wistar rats were divided into four groups: control and groups treated during 30 days with 100, 200 and 400 mg of lyophilized aqueous extract of *S. buxifolia* (SBSB)/kg of body weight. This study was planned to explore hepatotoxic effect of SBSB, which was assessed by serum transaminases (ALT and AST). Thiobarbituric acid reactive substances (TBARS) levels were determined in liver, along with thiols content (NPSH), catalase (CAT) activity and, superoxide dismutase (SOD) enzymes. Histopathological studies of liver tissue were performed. Flavonoids and phenolics were quantified in SBSB by high performance liquid chromatography with diode array detection (HPLC/DAD). We did not observe alterations on redox status (TBARS, NPSH, CAT and, SOD) in the control and experimental groups. An increase on AST activity was only observed at 200 mg of SBSB, whereas ALT score was not affected by SBSB. Moreover, no morphological alterations were observed on the hepatocytes, matching the analysed biochemical parameters. This way, we conclude that SBSB was not toxic.

## 1. Introduction

In the last few years, there was an explosion in the number of people taking herbal and over-the-counter alternative medicines, principally in Western countries [1]. Many reasons lead people to choose to take herbal remedies, like the side-effects of prescription drugs, which are widely publicized. Herbal preparations are perceived as safe, and therefore innocuous. However, several herbal medicines are reported to have hepatotoxic effects. Although the hepatotoxicity of herbal remedies is noted in case reports, this toxicity also could be due by contaminants, adulterants, confusion in identification and seasonal variations of plant composition [2], therefore, the safety of any herbal preparation cannot be predicted unless it is tested scientifically. In some patients the liver disorders are caused by indiosyncratic reactions to a certain compound but there are some hepatotoxic phytochemicals such as pyrrolizidine alkaloids, furanoneoclerodane diterpenoids and lignans that are well documented [3,4,5]. Case reports of liver complications related to the consumption of “kava” (*Piper methysticum*), “cascara sagrada” (*Rhamnus purshiana*), “germander” (*Teucrium chamaedrys*), “senna” (*Cassia acutifolia*), “valeriana” (*Valeriana officinales*), “sacaca” (*Croton cajucara*) and, “comfrey” (*Symphytum officinale*) are cited in the literature [6,7]. These plants may transiently elevate conjugated bilirrubin, asparate aminotransferase (AST), alanine aminotransferase (ALT), γ-glutamyltransferase (GGT), and alkaline phosphatase (AF) levels in serum of herbal consumers. Patients that undergo chronic treatment with medicinal plants can manifest cholestatic hepatitis and liver failure; this is well demonstrated by the indiscriminate use of comfrey, which has led to several hepatic injuries [7]. There have also been various reports describing its toxic features in South Africa [8]. The most common clinical evidence of hepatic disorders in patients who consume comfrey “bush tea” is venooclusive disease [9]. The pyrrolizidine alkaloids from comfrey can produce a disorder similar to Budd-Chiarri syndrome; and, in Boston (MA, USA), 11% of reported cases of Buss-Chiari syndrome were attributed to this herbal [10].

*Scutia buxifolia* Reissek (Rhamnaceae) is widely available in Argentina, Uruguay and Brazil [11]. It is a small prickly tree, commonly known as “coronilha”, whose stem bark infusion has diuretic and hypotensive properties [12]. Phytochemical screening of the leaves and stem bark of *S. buxifolia* crude extract and its fractions (butanolic, ethyl acetate and dichlorometane) revealed the presence of flavonoids, alkaloids, phenolics, and tannins [13]. Four quercetin-type flavonoids were isolated: quercetin, quercitrin, isoquercitrin, and rutin, being quercetrin the major component present in the ethyl acetate fraction [14]. Cycloptides alkaloids isolated as scutianines from methanolic root bark extract of *S. buxifolia* display antimicrobial activity against Gram (+), Gram (−) bacteria, and yeasts [15]. The analgesic effect of scutianine B was investigated by a tail flick test and it showed antinociceptive action at 15 and 60 min after administration [16].

Several studies have addressed the antioxidant properties of *S. buxifolia*, and some its biological actions, but little is known about its toxicicity [12]. Previous data revealed a cytotoxic activity of extracts and fractions prepared from different parts of *S. buxifolia* in a brine shrimp (*Artemia salina*) lethality bioassay. In this report, all tested plant fractions were toxic for *A. salina* [17]. The acute toxicity assay of single doses of *S. buxifolia* lyophilized aqueous extract (SBSB) at different concentrations was done by Freitas *et al.* [18]; these authors speculate that the lethal dose of SBSB is higher than 400 mg/kg body weight because the tested doses did not cause any mortality in animals [18].

In the present communication, we planned to explore for the first time the putative hepatotoxic effect of SBSB in animals treated sub-chronically, which was assessed by hepatic damage markers such as serum transaminases (ALT and AST). The hepatic redox balance was determined by thiobarbituric acid reactive substances (TBARS), antioxidant enzymes and, quantification of non-protein sulfhydryl groups (NPSH) tests, along with liver histopathological studies. Phenolic and flavonoids were quantified by High Performance Liquid Chromatography-Diode Array Detector (HPLC-DAD) analysis of SBSB.

## 2. Results

### 2.1. Quantification of Phenolic and Flavonoids Compounds by HPLC-DAD

**Figure 1:**
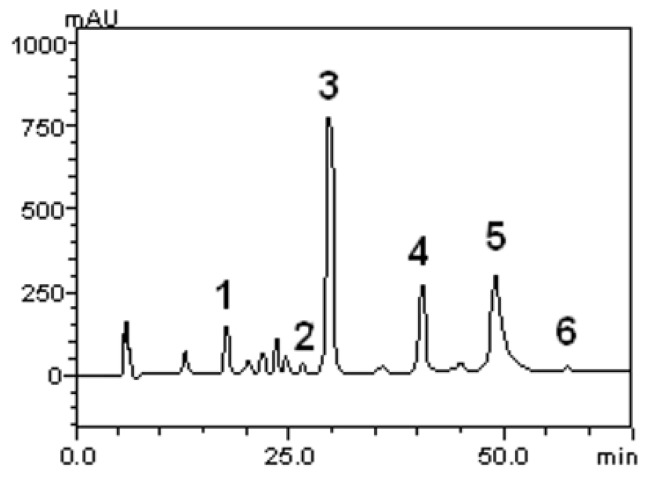
Representative high performance liquid chromatography profile of the *S. buxifolia* lyophilized extract. Gallic acid (1), chlorogenic acid (2), caffeic acid (3), rutin (4), quercetin (5) and kaempferol (6).

Flavonoids, such as quercetin, rutin and kaempferol, along with caffeic, gallic, and chlorogenic acids were quantified. The HPLC-DAD profile reveled a high concentration of caffeic acid in comparison to the other compounds. The concentration for each substance is given in Table 1. Also, we show the corresponding chromatogram in Figure 1.

**Table 1:** Phenolics and flavonoids composition of SBSB.

Component	Extract	
	mg/g *	%
Gallic acid	51.88 ± 0.01 a	5.18
Chlorogenic acid	3.98 ± 0.19 b	0.39
Caffeic acid	247.21 ± 2.17 c	24.72
Rutin	11.36 ± 0.63 d	11.16
Quercetin	132.59 ± 12.83 e	13.25
Kaempferol	1.75 ± 0.01 f	0.17

* Results are expressed as mean ± SD of three determinations. Averages followed by different letters differ by Tukey test at *p* < 0.05.

### 2.2. Biochemical Parameters in Blood

The serum transaminases (ALT and AST) were chosen to analyze the hepatic function of animals treated with SBSB at different doses, because they are more sensitive to liver damage. The quantification of these parameters occurred on the 15th and 30th day of treatment.

**Figure 2:**
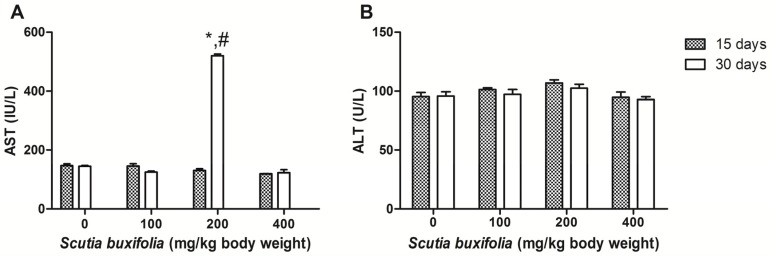
Asparate aminotransferase (AST) activity (**A**); alanine aminotransferase (ALT) activity (**B**).

The control group showed 147.0 ± 6.06 U/L and 95.3 ± 8.4 U/L for AST and ALT, respectively, on 15th day. On the 30th day of treatment for the control, the AST and ALT activities were 144.58 ± 8.11 U/L and 95.75 ± 9.33 U/L, respectively. ANOVA for repeated measures showed that there were no differences between days for this group on AST and ALT scores. Statistical analysis revealed that sub-chronic treatment with 100 mg of SBSB/kg body weight did not change the transaminases during the experimental protocol (Figure 2). We observed that the animals treated with 200 mg of SBSB/kg body weight did not show fluctuations on ALT between days. However, the AST value was increased 3.7 times on the 30th day (519.5 ± 6.51 U/L) in comparison to the 15th day (130.0 ± 6.28 U/L) (*p* < 0.05) for the animals treated with this dose (Figure 2A). The higher dosage (400 mg of SBSB/kg body weight) did not alter the evaluated parameters (Figure 2).

* Different between days; # Different from control group on the same day (p < 0.05). Data are expressed in means ± S.D.

### 2.3. Effect of *S. buxifolia* on Lipid Peroxidation

**Figure 3:**
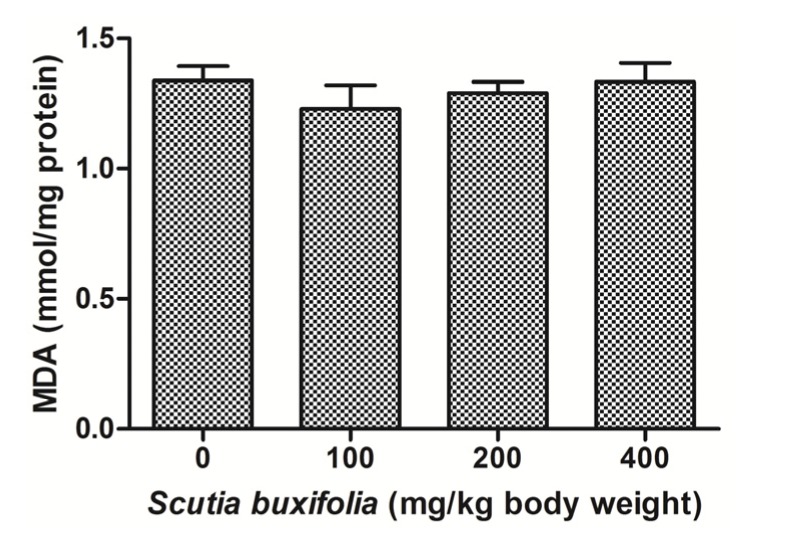
Thiobarbituric acid reactive substances (TBARS) levels expressed in nmol MDA/mg protein. No significant difference was noted between control group and 100, 200, 400 mg of SBSB/kg body weight treated groups (*p* < 0.05). Data are expressed in mean ± SD.

The level of MDA in liver tissue determined by a thiobarbituric acid reactive substances (TBARS) assay was not affect by the treatment with SBSB at different dosages compared to control group (Figure 3). MDA content for control group and groups treated with 100, 200 and 400 mg SBSB/kg body weight were 1.33 ± 0.11, 1.23 ± 0.18, 1.29 ± 0.10, and 1.34 ± 0.21, respectively. 

### 2.4. Effect of *S. buxifolia* on Tissue Sulfhydryl Groups (NPSH)

**Figure 4:**
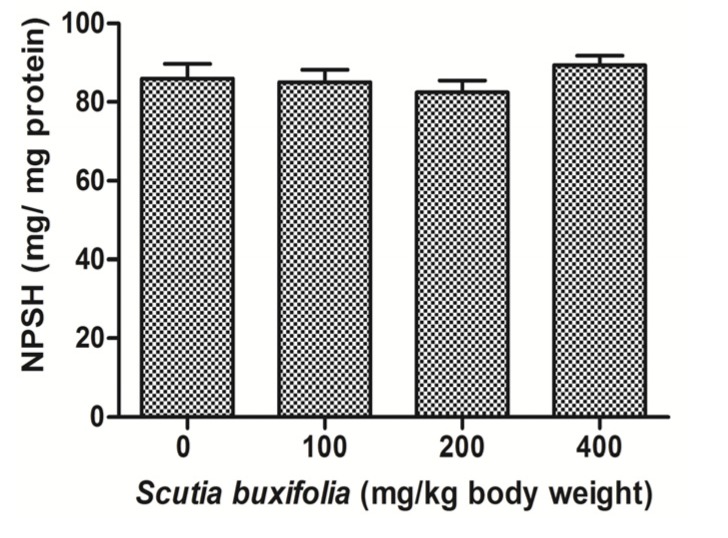
Tissue sulfhydryl groups content (NPSH). No significant difference was noted between control group and 100, 200, 400 mg of SBSB/kg body weight treated groups (*p* < 0.05). Data are expressed in mean ± SD.

The level of NPSH in liver tissue was not affected by the treatment with SBSB at different dosages compared to control group (Figure 4). NPSH content for control group and treated groups with 100, 200 and 400 mg SBSB/kg body weight were 85.95 ± 9.10, 85.0 ± 7.83, 82.49 ± 7.18, and 89.33 ± 6.09, respectively. 

### 2.5. Effect of *S. Buxifolia* on Enzymatic Antioxidant Defense

**Figure 5:**
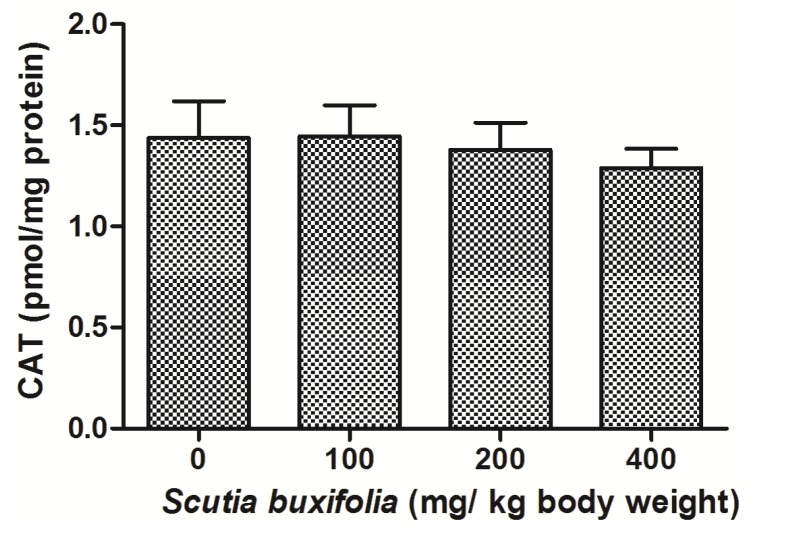
Catalase (CAT) activity. No significant difference was noted between control group and 100, 200, 400 mg of SBSB/kg body weight treated groups (*p* < 0.05). Data are expressed in mean ± SD.

**Figure 6:**
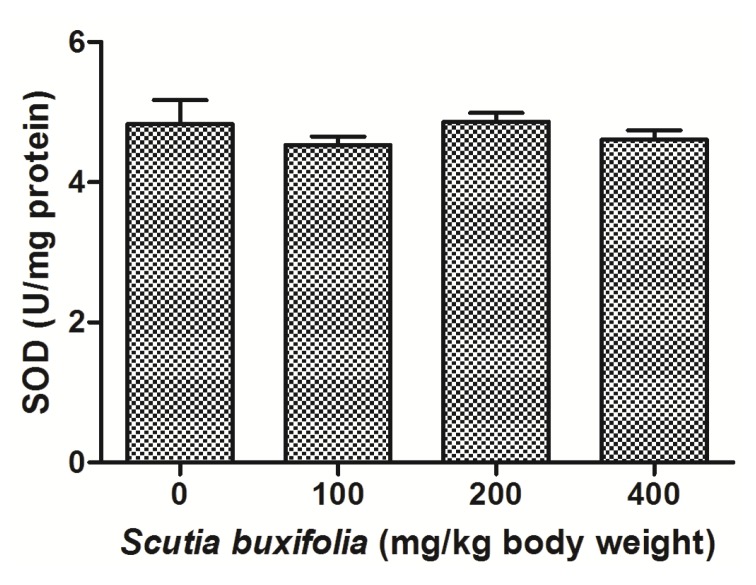
Superoxide dismutase (SOD) activity. No significant difference was noted between control group and 100, 200, 400 mg of SBSB/kg body weight treated groups (*p* < 0.05). Data are expressed in mean ± SD.

Catalase (CAT) activity is responsible for decomposition of hydrogen peroxide in oxygen and water. There were no differences between groups for CAT activity (Figure 5). Superoxide dismutase (SOD) activity was not affect by sub-chronic treatment with SBSB at different dosages (Figure 6). 

### 2.6. Histopathological Studies

**Figure 7:**
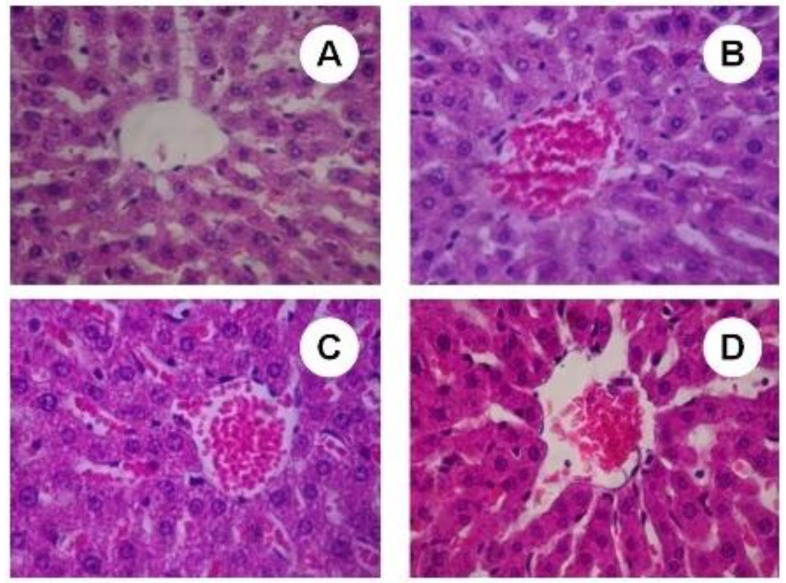
Light micrograph (H & E staining) of liver. (**A**) control group (400×); (**B**) 100 mg of SBSB/weight treated group (400×); (**C**) 200 mg of SBSB/weight treated group (400×); (**D**) 400 mg of SBSB/weight treated group (400×). Figures show the centrilobular vein. There is presence of red blood cells on (C) and (D).

The sub-chronic treatment with SBSB at different dosages evaluated on present protocol did not induced negative features in hepatocytes morphology. All sections analyzed received score 0 (Figure 7). 

## 3. Discussion

Herbal medicine is the base of Traditional Chinese Medicine and Aiurvedic Medicine [5]. The massive consumption of medicinal plants has increased the cases of intoxication by infusions, decoctions, and herbal remedies. In Brazil, some plants which possess depurative, hypoglycaemic and diuretic effects were reported to cause hepatic disorders such as cirrhosis, acute hepatitis and, fulminating hepatitis [6]. The hepatotoxic effects of these plants have a common basis: (1) overabundance of toxic secondary metabolites; (2) toxicity when consumed for a long time and; (3) increase of free radical production [1]. 

*Scutia buxifolia* Reissek is widely used for the treatment of hypertension, renal dysfunction and, weight loss purposes [19]. However, there is little knowledge about its biological activities and toxic effects. Various studies were carried out in order to determine the total antioxidant capacity of *S. buxifolia* crude extract and its fractions [11,20]. The antioxidant scavenger capacity measured by the DPPH method revealed that the aqueous extract used in this protocol has an antioxidant power similar to that of ascorbic acid, a well-known antioxidant [18]. The HPLC/DAD fingerprint quantified phenolics and flavonoids present in *S. buxifolia* aqueous extract. Some modifications to the HPLC methodologies allowed the quantification of kaempferol. Previous data did not show the presence of this flavonoid in this extract [18].

Gallic, chlorogenic, and caffeic acids, along with rutin, and quercetin, were quantified (Figure 1 and Table 1). These substances are great antioxidants because in their molecular structures contain hydroxyl groups. Quercetin, one of the most common flavonoids in various botanicals, has been studied as a potent hepatoprotective substance [21]. 

Plants can develop hepatotoxicity because the liver is susceptible to external agents, mainly chemical ones and, various herbal remedies generate an imbalance in some damage markers such as enzymes. ALT enzyme is a more sensitive acute hepatotoxicy marker than AST, because while the first enzyme is essentially hepatic, the second also can be found in high concentrations in other organs such as kidney, lungs and heart [22].

The ALT and AST activities remained unchanged during the experimental protocol in the control group, 100 and 400 mg of SBSB/kg body weight-treated groups (Figure 2). The second dose tested (200 mg of SBSB/kg body weight) did not cause changes on ALT score between days and, in comparison to control group (Figure 2B), however there was a remarkable elevation in serum AST enzymatic activity on the 30th day (*p* < 0.05) (Figure 2A). This fact might not be directly related to SBSB toxicity because transaminase activity results are influenced by haemolysis, muscular stress and, xenobiotics like anesthetics [23].

Researchers in toxicity assays, beyond the use classical markers including transaminases, have evaluated the redox state of organs by antioxidant activity enzymes and measurements of macromolecule oxidation [24]. In the present study, the oxidative damage on cellular membranes was accessed by a thiobarbituric acid reactive substances (TBARS) assay. This assay quantified the malondialdehyde (MDA) which is a product of lipoperoxidation (LPO). *S. buxifolia* did not cause overproduction of free radicals in the liver because the MDA levels did not differ among experimental and control groups (Figure 3). SBSB controlled the level of oxidants, probably due the presence of natural antioxidant compounds. These compounds, previously reported, have been investigated extensively and there is evidence that they are involved on scavenging of radicals as well as indirect activation of transcription factors that regulate the expression of genes encoding for antioxidant enzymes [25].

Tissue sulfhydryl groups act against reactive oxygen species (ROS) which are related to toxic hepatitis induced by herbs. The principal NPSH, comprising 75%–90% of total intracellular NPSH, is reduced glutathione (GSH) [26]. GSH plays an important role in antioxidant defense, because it possesses not only direct radical-scavenging properties, but it also an essential component of glutathione peroxidase (GPx) systems, which eliminate different hydroperoxides [27].

Toxic plants investigated in sub-chronic or chronic assays, deplete the GSH content present in liver due principally the pro-oxidant effects of extracts at higher doses and to overcome this free-radical stress, GSH is being utilized as the first line of defense [24]. Concerning non-enzymatic defenses, there are no significance changes in thiols content (NPSH), certainly because a synergistic antioxidant effect occurs between NPSH content and SBSB phytochemicals in the liver tissue (Figure 4). 

The CAT and SOD antioxidant activities were also evaluated in hepatic tissue of animals treated of SBSB. CAT together with SOD, and GPx constitute the primary enzymatic defense, catalyzing decomposition of ROS. We noted that sub-chronic SBSB treatment did not alter CAT and SOD activities in experimental groups, comparing to control (Figure 5 and Figure 6, respectively) [28]. Theoretically, activity of SOD and CAT should be unaffected or rise as a compensatory mechanism to overcome free-radical stress [29]. 

Several studies have shown that increased oxidative stress and inflammation play a crucial role in tissue alterations. In the hepatotoxicity, activated Kupffer cells (resident macrophages and primary immune cells of the liver) and infiltrating neutrophils are major sources of ROS and, proinflammatory cytokines, which promote oxidative stress [30,31,32,33,34].

Although we observed an increase on the AST value for 200 mg of SBSB/kg body weight, there were no observed negative features at a microscopic level. Histopatological analysis of the experimental and control groups showed an absence of hepatocellular necrosis, cirrhosis, inflammation, sinusoidal congestion and, central vein disruption. In Figure 7A–D the centrilobular vein in liver sections of control and treated groups with 100, 200, and 400 mg of SBSB/kg body weight, respectively, appears. Xenobiotics such toxic plants modify the hepatocytes architecture by redox imbalance, and in this protocol SBSB preserved the hepatic tissue. 

## 4. Experimental

### 4.1. Plant Material and Extract Preparation

Stem bark of *S. buxifolia* was collected in Dom Pedrito (Rio Grande do Sul State of Brazil) in February of 2011 (coordinates 30°59′09″S and 54°27′44″W). A dried voucher specimen is preserved in the herbarium of the Department of Biology at Federal University of Santa Maria under register number SMBD 10919. The plant parts were dried at room temperature and powdered in a knife mill. The powdered stem bark (300.5 g) was used for preparation of an infusion with one liter of boiling water. After filtration, the infusion was evaporated under reduced pressure to remove the water. The infusion was submitted to lyophilisation. This extract was dissolved in distilled water for the biological assays.

### 4.2. Chemicals

All chemical were of analytical grade. Methanol, acetic acid, gallic acid, chlorogenic acid and caffeic acid were purchased from Merck (Darmstadt, Germany). Quercetin, rutin and kaempferol were acquired from Sigma Chemical Co. (St. Louis, MO, USA).

### 4.3. Quantification of Phenolics and Flavonoids Compounds by HPLC-DAD

High performance liquid chromatography (HPLC-DAD) was performed with a Shimadzu HPLC system (Kyoto, Japan), equipped with a Prominence Auto Sampler (SIL-20A), and Shimadzu LC-20AT reciprocating pumps connected to a DGU 20A5 degasser and a CBM 20A integrator, SPD-M20A DAD (diode array) UV-VIS detector and LC solution 1.22 SP1 software. Reverse phase chromatographic analyses were carried out under gradient conditions using a C_18_ column (4.6 mm × 250 mm) packed with 5 μm diameter particles; the mobile phases were water containing 2% acetic acid (A) and methanol (B), and the composition gradient was: 5% (B) for 2 min; 25% (B) until 10 min; 40, 50, 60, 70, 80 and 100% (B) every 10 min, following the method described by Evaristo and Leitão with slight modifications [35]. The sample and mobile phase were filtered through a 0.45 μm membrane filter (Millipore) and then degassed by an ultrasonic bath prior to use. Stock solutions of reference standards were prepared in the HPLC mobile phase at a concentration range of 0.020–0.200 mg/mL quercetin, rutin and kaempferol, and 0.010–0.100 mg/mL for gallic, chlorogenic and caffeic acids. Quantification was carried out by integration of the peaks using the external standard method at 254 nm for gallic acid, 327 nm for chlorogenic and caffeic acids, and 365 nm for quercetin, rutin and kaempferol. The flow rate was 0.7 mL/min and the injection volume was 40 μL. The chromatography peaks were confirmed by comparing their retention times with those of reference standards and by their DAD spectra (200 to 500 nm). Calibration curve for gallic acid: Y = 53985x + 1028.8 (r = 0.9997); chlorogenic acid: Y = 287603x + 1093 (r = 0.9989); caffeic acid: Y = 13745x + 1281.7 (r = 0.9998); rutin: Y = 14791x + 1264.5 (r = 0.9998); quercetin: Y = 88513x + 5548 (r = 0.9999) and kaempferol: Y = 51285x − 1063.9 (r = 0.9992). All chromatography operations were carried out at ambient temperature and in triplicate.

### 4.4. Animals

Thirty-two male Wistar rats (200–250 g), obtained from the General Animal House of the Federal University of Santa Maria, were kept in a separate animal room, in a 12 h light/dark cycle at room temperature and were feed *ad libitum* with free access to tap water. All animals were used according to of the Committee on Care and Use of Experimental Animal Resources (CEUA) from Federal University of Santa Maria, Brazil (project number 091-2011). In addition, efforts were made to minimize animal suffering and reduce the number of animals used.

### 4.5. Experimental Design

In the sub-chronic study, thirty-two rats were randomly divided into four groups. Treatment groups were based on previous study with some modification as shown as below:

1. Group I: Received distilled water (0 mg of SBSB/kg body weight).
2. Group II: Received SBSB at a concentration of 100 mg/kg body weight.
3. Group III: Received SBSB at a concentration of 200 mg/kg body weight.
4. Group IV: Received SBSB at a concentration of 400 mg/kg body weight.

Distilled water (vehicle) and the extract at different dosages were administered by gavage (0.5 mL/kg body weight). The animals were treated in the morning daily, during 30 days. The dosages of SBSB used in the present protocol were selected according Freitas *et al.* [18]. 

### 4.6. Liver Samples

Twenty-four hours after the latest dosage of SBSB, the animals were euthanized by deep anesthesia induced by thiopental at 100 mg/kg body weight, administered intraperitoneally. The liver was excised and immediately washed with saline solution (NaCl 0.9%) and 1 g of the tissue was homogenized in 9 volumes of potassium sodium buffer 0.1 M, pH 7.4 using a Polytron mixer (Kinematica AG, Luzern, Switzerland). A liver sample was collected for histopathological analysis.

### 4.7. Measurement of Biochemical Parameters in Blood

Blood was collected by retro-orbital puncture in a plain tube for serum biochemistry [36]. Blood samples were collected from each rat on the 15th and 30th day and the procedure occurred at morning 24 h after the last dose extract administration. For hepatic damage and function analysis, serum alanine aminotransferase (ALT) and, asparate aminotransferase (AST) activities were determined. The assays were performed in a Cobas Mira^®^ automatic analyser (Roche Diagnostics. Basel, Switzerland) with Bioclin^®^ commercial systems.

### 4.8. Thiobarbituric Acid Reactive Substances

Liver tissue lipoperoxidation (LPO) estimation was performed using the TBARS assay as previously described, where the colorimetric reaction of the LPO product malondialdehyde (MDA) with thiobarbituric acid (TBA) is quantified. This reaction produces a colored compound that absorbs maximally at 532 nm [37].

### 4.9. Non-Enzimatic Antioxidant Defense

Tissue sulfhydryl groups (NPSH) was quantified after mixing the homogenate with 10% trichloroacetic acid (1:1, v/v), followed by centrifugation, as described by Ellman. Cysteine was used for preparation of a standard curve [38].

### 4.10. Catalase Activity

Catalase (CAT) activity was determined by measuring the decrease in hydrogen peroxide (H_2_O_2_) absorption at 32 °C. The method is based on the consumption of H_2_O_2_ by CAT and loss of absorbance at 240 nm [39].

### 4.11. Superoxide Dismutase Activity

Superoxide dismutase is an enzyme which catalyzes the dismutation of superoxide radical to form hydrogen peroxide and oxygen. The assay for determination of indirect SOD-activity is based in the inhibition of reaction between superoxide radical with adrenaline [40].

### 4.12. Histopathological Studies

For the histopathological studies, samples of the liver from control and experimental groups were fixed in 10% buffered formalin for 24 hours. The processed tissues were embedded in paraffin blocks and sections made were stained with hematoxilin and eosin (H & E) dye. The sections were analyzed by observing under light microscope at 400× magnification. These sections were examined by a pathologist without knowledge of the experimental groups for presence of negative features. Preparations were scored for hemorrhage, inflammation, cirrhosis, necrosis, sinusoidal congestion and disruption of central vein. An arbitrary scale was created for analyze the sections: Tissue with absence of negative features received score 0. Liver tissue with minor alterations was given a score of +1. Those with moderate and severe negative features were given a score of +2 and +3, respectively [41]. 

### 4.13. Statistical Analysis

The results were expressed as mean ± standard deviation (SD). Statistical comparisons were performed by one-way analysis of variance followed Tukey’s *post-hoc* test. The data were analyzed by using Statistical Package for the Social Sciences (SPSS, version 18.0). A *p*-value less than 0.05 were considered to be significant different.

## 5. Conclusions

This brief report about *S. buxifolia* sub-chronic toxicity revealed elevations in AST levels in 200 mg of SBSB/kg body weight treated groups which may be associated to haemolysis and not necessary related to any SBSB component. This hypothesis is supported by the unchanged parameters for the other groups. Apparently, SBSB are safe to hepatocytes because we did not observe any negative features in our histopathological studies. In addition, the redox state of SBSB-dosed groups, assessed by TBARS, NPSH, SOD and CAT tests, was very similar to the control group. In conclusion, SBSB was revealed to be non-toxic to the liver. 
